# Clinical characteristics and surgical treatment of congenital gluteal dermal sinus tract in children: a 15-year retrospective single-center clinical experience

**DOI:** 10.3389/fped.2026.1836614

**Published:** 2026-05-08

**Authors:** Debao Li, Shan Zheng, Xianmin Xiao, Yangyang Ma, Qingchi Zhang, Chenbin Dong, Weijing He, Gong Chen, Chun Shen, Song Sun

**Affiliations:** 1 Department of Pediatric Surgery, Children’s Hospital of Fudan University, National Children’s Medical Center, Shanghai, China; 2 Neonatal Disease, Ministry of Health, Shanghai Key Laboratory of Birth Defects, Shanghai, China; 3 Department of Pathology, Children’s Hospital of Fudan University, National Children’s Medical Center, Shanghai, China; 4 Children’s Hospital of Fudan University (Xiamen Branch), Xiamen Children’s Hospital, Xiamen, China; 5 Xiamen Key Laboratory of Pediatric General Surgery Diseases, Children’s Hospital of Fudan University (Xiamen Branch), Xiamen Children’s Hospital, Xiamen, China

**Keywords:** congenital gluteal dermal sinus tract, imaging diagnosis, pediatrics, prognosis, surgical resection

## Abstract

**Objective:**

To summarize the clinical features, diagnosis, treatment, and prognosis of congenital gluteal dermal sinus tracts in children, providing insights for better clinical management.

**Methods:**

A retrospective analysis was conducted on 20 patients diagnosed with congenital gluteal dermal sinus tracts at the Children's Hospital of Fudan University over 15 years. The analysis focused on age, gender, diagnostic delay, diagnostic methods, course and opening of the sinus tract, and postoperative follow-up.

**Results:**

The study included 6 males and 14 females, with symptom onset ranging from birth to 12 years (median age of 9.5 months). Initial symptoms included abnormal gluteal depression with recurrent infections (*n* = 8), unexplained recurrent infections (*n* = 6), and asymptomatic gluteal depression or small holes (*n* = 6). The time from symptom onset to diagnosis ranged from 1 to 102 months (median: 13.5 months). MRI was positive in 87.5%, identifying the tract in 81.25%. CT scans were positive in all cases, but only 50% identified the tract. Sinus tractography successfully identified the tract in 7 of 10 patients. The sinus tracts were classified into three types based on the opening location. All sinus tracts were excised during surgery, with an average length of 4.95 cm. Two patients experienced recurrence and underwent reoperation, while the remaining 15 had no recurrence.

**Conclusion:**

Congenital gluteal dermal sinus tracts are more common in females and often misdiagnosed. CT is more sensitive but less specific than MRI. Sinus tractography helps determine the tract's path. Prognosis is generally good after complete excision, with recurrence being the main postoperative complication.

## Introduction

Congenital gluteal dermal sinus tract is a rare condition observed in children, often misdiagnosed due to its atypical presentation (1–3). Unlike the congenital dermal sinus tract, which typically occurs along the midline and is often associated with the central nervous system (4, 5), congenital gluteal dermal sinus tracts are usually found in off-midline regions, such as the buttock or posterior perianal area (6–9). Because they rarely extend into the spinal canal, neurological complications are uncommon (10). Clinically, affected children often present with a congenital dimple or small pit in the gluteal region, usually complicated by recurrent infections or sinus tract formation (6–16).

A review of the available literature reveals that only a few dozen cases of congenital gluteal dermal sinus tracts have been reported worldwide (6–16). Moreover, these reports are mostly isolated case descriptions or small case series, highlighting a lack of comprehensive research on the clinical classification, diagnostic criteria, and long-term outcomes of this condition.

In this study, we retrospectively reviewed 20 pediatric cases of congenital gluteal dermal sinus tract treated at the Children's Hospital of Fudan University over the past 15 years. The aim was to summarize the clinical features, diagnostic strategies, treatment experiences, and outcomes of this rare disease, providing valuable insights for improving diagnosis, management, and long-term care.

## Methods

### Patients

This study used a retrospective design and included children who underwent surgical treatment at the Children's Hospital of Fudan University between January 2009 and December 2023 and were diagnosed with congenital gluteal dermal sinus tracts. The diagnosis was established based on a combination of clinical presentation, imaging evaluation, intraoperative findings, and postoperative pathological confirmation. Clinical manifestations mainly included recurrent infection, local swelling, persistent or intermittent discharge, and cutaneous dimples or sinus openings in the gluteal or posterior perianal region. Imaging examinations, including ultrasound, MRI, and/or CT when available, were used to evaluate the extent of the lesion and its relationship with adjacent structures. Definitive diagnosis was confirmed by surgical findings and postoperative pathological examination. The inclusion criteria were as follows: 1) Patients who sought treatment between 2009 and 2023; 2) Patients who underwent surgical treatment at the Children's Hospital of Fudan University; 3) patients with a confirmed diagnosis of congenital gluteal dermal sinus tract based on the above clinical, imaging, intraoperative, and pathological findings. The exclusion criteria were: 1) Incomplete medical records; 2) No surgical treatment; 3) Unclear pathological diagnosis. In total, 20 eligible children were included in the study.

The following data were retrospectively collected: age, gender, initial symptoms, first visit time, diagnostic delays, diagnostic methods, pathological findings, opening location and course of the sinus, and postoperative follow-up data. Clinical data were obtained from electronic medical records, and follow-up information was collected through outpatient visits or telephone interviews with the patients’ families. A standardized data collection form was used during follow-up to improve the consistency of the information recorded, including postoperative recovery, wound healing, symptom recurrence, and other relevant clinical outcomes. This study was approved by the Ethics Committee of the Children's Hospital of Fudan University. Written informed consent was obtained from the patients’ parents or legal guardians.

### Data analysis

Statistical analysis was performed using SAS version 9.2 software (SAS Institute, Cary, NC, USA). Continuous variables are presented as mean ± standard deviation or median (range), as appropriate. Categorical variables are presented as frequencies and percentages. Given the small sample size and the descriptive nature of this retrospective case series, the analysis was primarily descriptive, and no formal inferential statistical tests were performed.

## Results

### Demographic characteristics

A total of 20 children were included in this study, consisting of 14 females (70.0%) and 6 males (30.0%). The age at onset of the first symptoms ranged from 0 to 144 months, with a median age of 9.5 months. Nine children had symptoms detected at birth (0 months). The time from the onset of symptoms to diagnosis ranged from 1 to 102 months, with a median time of 13.5 months. The first symptoms were categorized into three types: 1) Recurrent gluteal infections in 6 cases (30.0%); 2) Gluteal depression with recurrent infections in 8 cases (40.0%); 3) Asymptomatic gluteal depression in 6 cases (30.0%). Regarding the number of gluteal depressions, 14 cases had a single gluteal depression (70.0%), while 6 cases had two depressions (30.0%). Five children had a history of prior surgery, with 2 having undergone sinus tract excision and 3 having had incision and drainage procedures. Regarding follow-up, 3 patients were lost in follow-up, while the remaining 17 patients were followed up for a period ranging from 5 months to 15 years, with a median follow-up time of 3.75 years. The prognosis was as follows: 15 patients were cured, and 2 patients experienced recurrent infections (Table 1).

**Table 1:** Basic information of patients.

No	Gender	Age at presentation	Delayed time of diagnosis	First symptom	Number of dimples	Previous operation	Prognosis
1	F	18	102	Recurrent gluteal infection	1	None	Cured
2	F	1	49	Recurrent gluteal infection	1	None	Cured
3	F	0	71	Gluteal depression with recurrent infection	2	Sinus tract excision	Recurrence
4	F	0	17	Gluteal depression with recurrent infection	1	None	Cured
5	M	16	10	Recurrent gluteal infection	1	Incision and drainage	Cured
6	F	15	12	Recurrent gluteal infection	1	None	Cured
7	F	132	48	Recurrent gluteal infection	1	Incision and drainage	Cured
8	M	144	12	Gluteal depression with recurrent infection	1	Incision and drainage	Recurrence
9	F	54	3	Gluteal depression without symptoms	2	None	Cured
10	M	22	23	Gluteal depression with recurrent infection	2	Sinus tract excision	Cured
11	F	0	9.6	Gluteal depression with recurrent infection	2	None	Cured
12	M	0	72	Recurrent gluteal infection	1	None	Cured
13	M	0	4	Gluteal depression without symptoms	1	None	Cured
14	F	10	2	Gluteal depression without symptoms	1	None	Cured
15	F	27	1	Gluteal depression with recurrent infection	1	None	Cured
16	F	0	25	Gluteal depression with recurrent infection	2	None	Cured
17	F	8	40	Gluteal depression without symptoms	1	None	Cured
18	M	11	7	Gluteal depression without symptoms	1	None	Cured
19	F	0	15	Gluteal depression with recurrent infection	2	None	Cured
20	F	9	2.7	Gluteal depression without symptoms	1	None	Cured

### Imaging inspection

**Figure 1:**
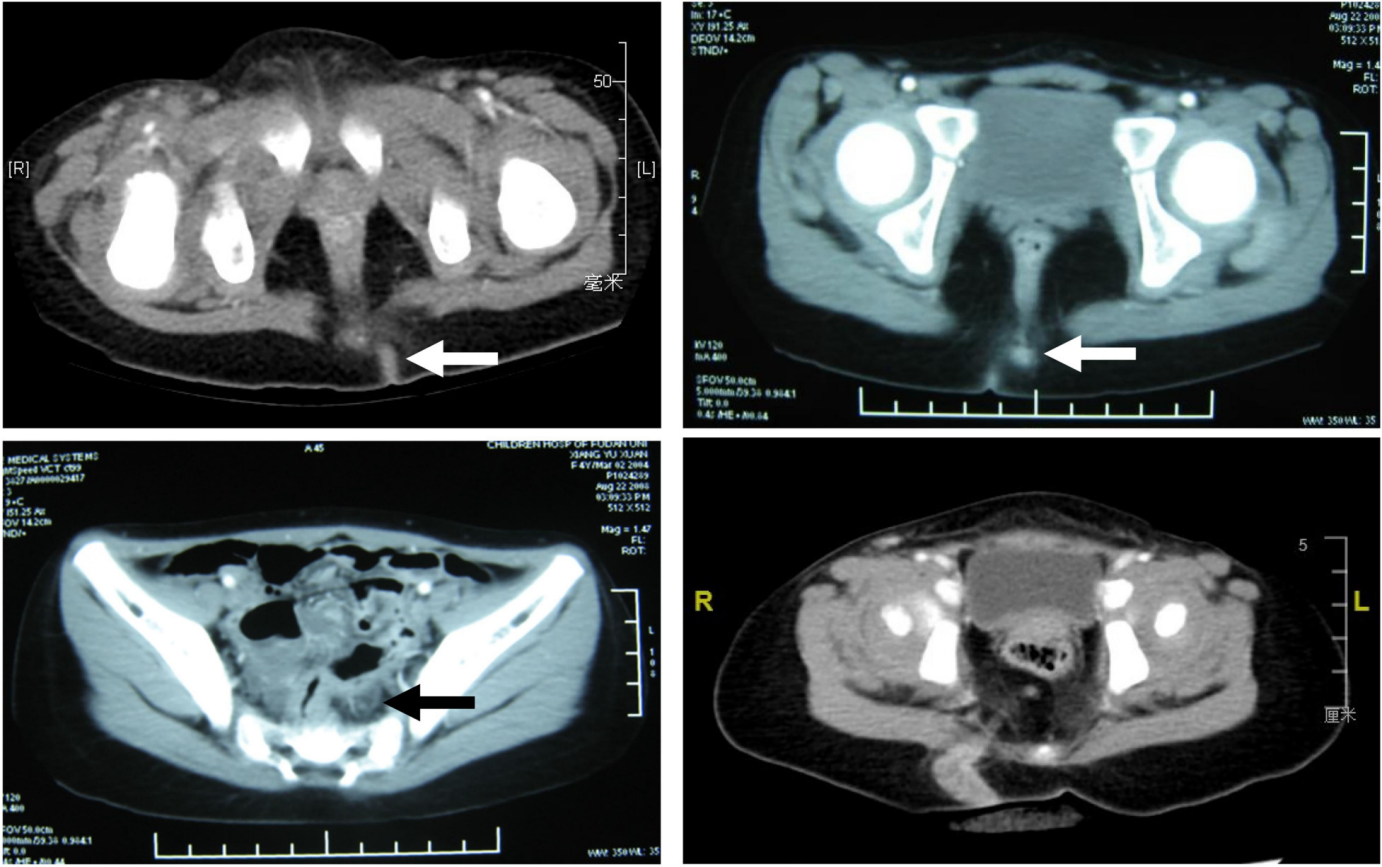
CT imaging of congenital gluteal dermal sinus tract: axial CT images of children with congenital gluteal dermal sinus tract, with arrows indicating the position of the fistula in different sequences.

**Figure 2:**
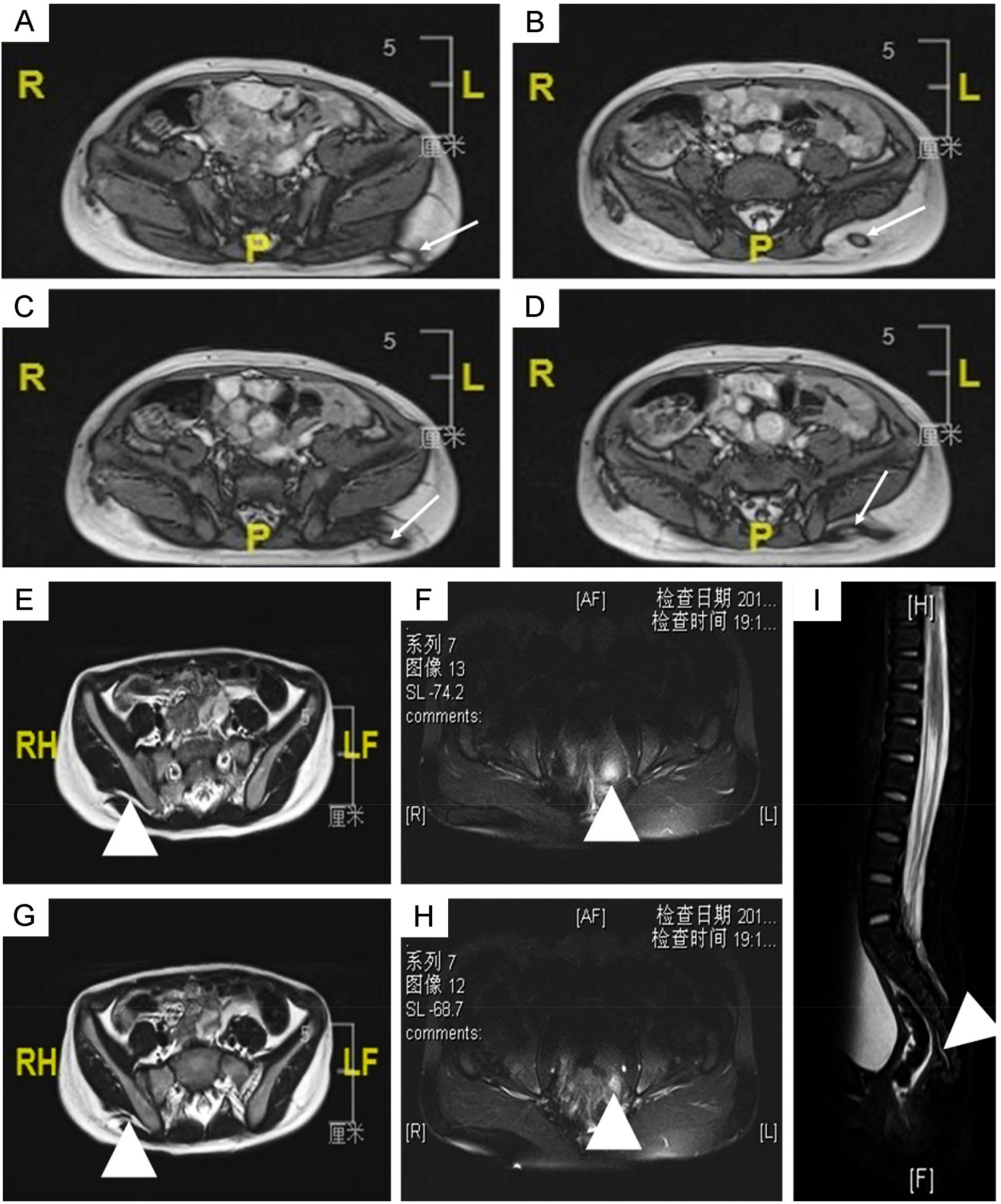
MRI imaging of congenital gluteal dermal sinus tract: **(A–H)**, axial MRI images of children with congenital gluteal dermal sinus tract, with arrows and triangles indicating the location of the fistula in different sequences; **(I)**, sagittal MRI image of children with congenital gluteal dermal sinus tract.

**Figure 3:**
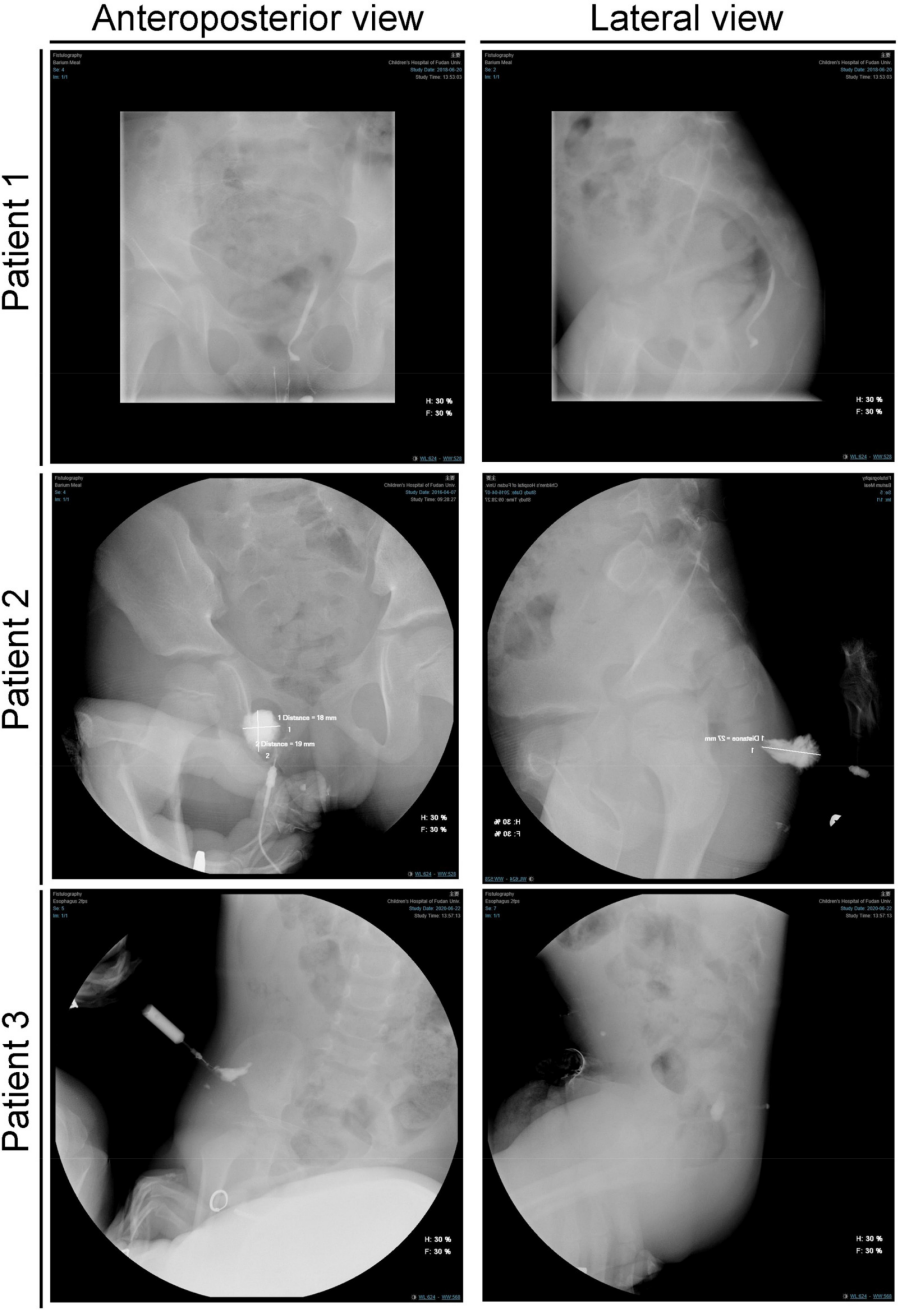
Fistulography of children with congenital gluteal dermal sinus tract in different views.

In this study, 8 children underwent CT scans (Figure 1), 16 children underwent MRI scans (Figure 2), and 10 children underwent fistulography (Figure 3). The sensitivity of the CT scans was 100% (8/8), with all 8 children showing positive findings (indicated by white and black arrows in Figure 1). However, only 4 cases showed the presence of a fistula, resulting in a specificity of 50% (Table 2).

**Table 2:** Imaging information.

Parameter	CT	MRI	Fistulography
No. of exams	8	16	10
Sensitivity	100%(8/8)	87.5%(14/16)	70%(7/10)
Specificity	50%(4/8)	81.25%(13/16)	70%(7/10)

Among the 16 children who underwent MRI, 14 showed positive findings (indicated by white and triangular arrows in Figure 2), giving a sensitivity of 87.5% (Table 2). Of these, 13 MRI reports identified the presence of a fistula, leading to a specificity of 81.25% (Table 2). In fistulography, 7 out of 10 children successfully underwent catheterization and imaging, which clearly showed the fistula and its course (Figure 3). Based on the imaging data, all fistulas were confirmed to be disconnected from the rectum and spinal canal.

### Fistula information

**Figure 4:**
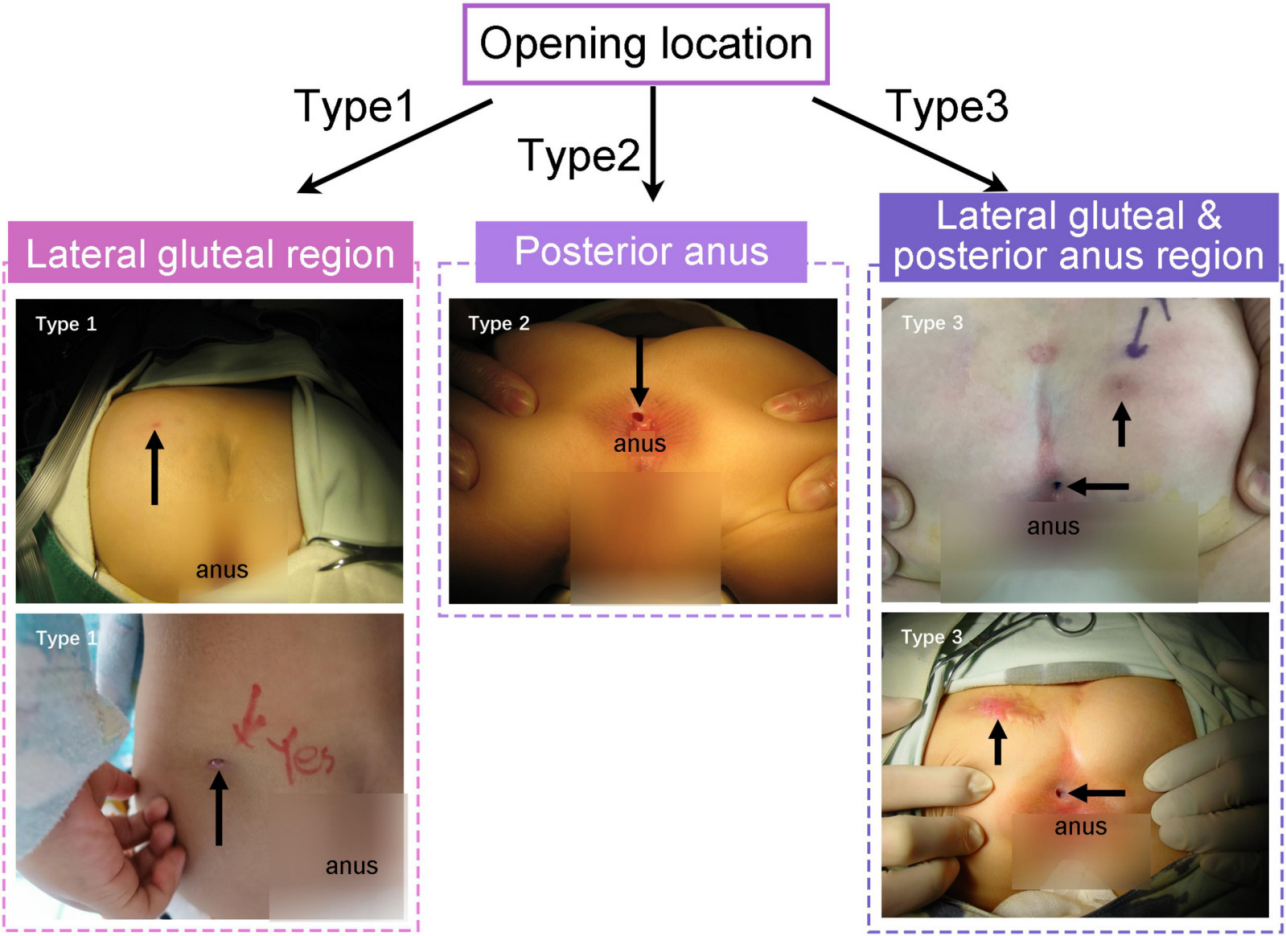
Classification of fistulas based on the location of the opening: black arrows indicate the location of the fistula opening.

Based on the location of the fistula opening, this study classified the children into three types (Figure 4): Type 1 as gluteal fistula (opening only in the gluteal region), Type 2 as posterior anal fistula (opening only posterior to the anus), and Type 3 as concurrent fistulas (openings at both the buttocks and posterior to the anus).

In Type 1, there were 7 cases, with gluteal fistulas located on the left side in 6 cases and on the right side in 1 case. In Type 2, there were 7 cases. In Type 3, there were 6 cases, with gluteal fistulas located on the left side in 2 cases and on the right side in 4 cases (Figure 4). Among the 6 cases in Type 3, 2 fistulas communicated internally, while 4 were connected by connective tissue through the presacral fascia (Table 3). All fistulas terminated in the fascia anterior or lateral to the sacrococcygeal region. The average length of the fistula was 4.95 ± 2.03 cm.

**Table 3:** Fistula Information.

Characteristic	Number
Opening position	
Buttocks (left side; right side)	*n* = 7 (6; 1)
Posterior anus	*n* = 7
Both (left side; right side)	*n* = 6 (2; 4)
Communicate with each other	*n* = 2
Divided by connective tissue through the presacral fascia	*n* = 4
Termination site	
Anterior or lateral sacrococcygeal fascia	*n* = 20
Contents of fistula	
Sebaceous-like material	*n* = 8
Infected tissue or purulent fluid	*n* = 6
Hair, oils, and other materials	*n* = 6

Regarding the contents of the fistulas, 8 cases contained sebaceous-like substances, 6 contained infected tissue or purulent fluid, and 6 contained hair, oils, and other materials.

### Treatment

**Figure 5:**
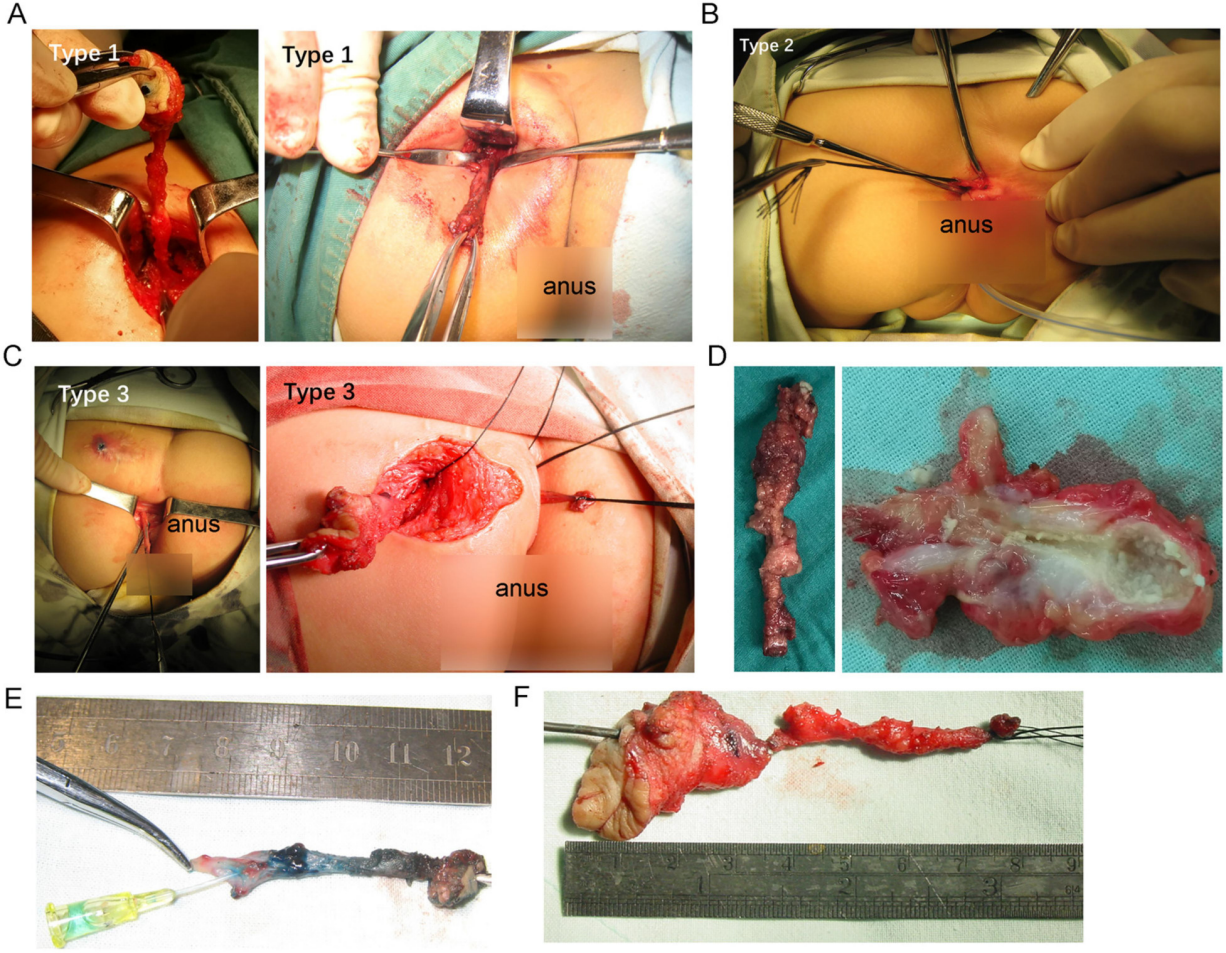
Intraoperative photos of three types of fistulas: **A-C**, intraoperative photos of children with type 1 to type 3 congenital gluteal dermal sinus tracts; **D**, gross view of the completely excised sinus tract; **E-F**, intraoperative photos using methylene blue to trace the fistula and the excised sinus tract.

All 20 children underwent surgical treatment. Preoperatively and intraoperatively, methylene blue was used to trace the fistulas, which were then completely excised along their course. The average length of the fistula was 4.95 ± 2.03 cm (Figure 5), and the fistulas contained hair, infected tissue, sebaceous-like material, and other substances. During the surgery, it was confirmed that none of the fistulas were connected to the rectum or sacral canal. One patient had an enlarged fistula that was closely adhered to the rectum, resulting in a rectal injury during the procedure. A stoma was created, and the patient successfully had the stoma closed 2 months later.

### Pathology

**Figure 6:**
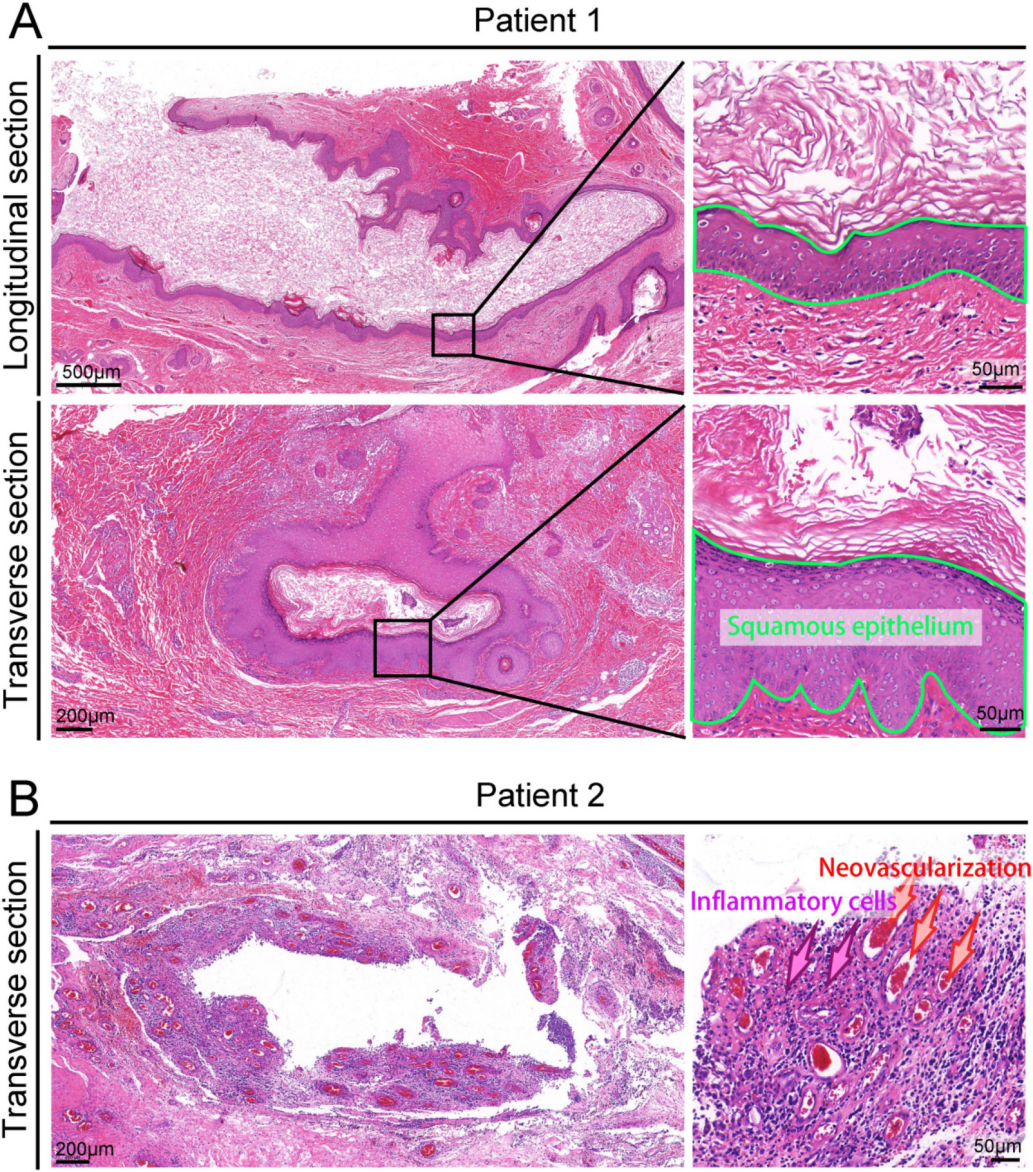
Fistula pathological phenotype. **(A)**, Cross-section and longitudinal section of the excised fistula stained with H&E, with the green circle indicating the presence of squamous epithelium lining; **(B)**, Cross-section of the fistula stained with H&E, showing numerous newly formed small blood vessels (red arrow) and inflammatory cells (purple arrow).

In terms of lumen structure, pathological slides from 13 children showed a lumen lined with squamous epithelium. In 2 cases, the slides indicated a lumen lined with stratified epithelium, and in 1 case, a lumen lined with ciliated columnar epithelium was observed. Three patients exhibited small blood vessel proliferation. Additionally, in 13 cases, the lumen indicated infiltration of inflammatory cells (Figure 6). Furthermore, skin appendages, keratinized material, hair, or muscle tissue were also found within the lumen.

### Prognosis

Seventeen patients were followed up for a period ranging from 5 months to 15 years, with a median follow-up time of 3.75 years. Two patients experienced recurrence 1 month post-surgery, and both underwent reoperation 1 year and 2 years after surgery, respectively, and were cured postoperatively. Both these two patients had a previous surgical history, one had a Type 2 fistula, and the other had a Type 3 fistula. One patient suffered rectal injury during surgery, which required the creation of a stoma; the stoma was closed 2 months later. No recurrence or signs of infection were observed in the remaining patients.

## Discussion

In the present study, congenital gluteal dermal sinus tract was classified into three types according to the number and location of the external openings. MRI played a central role in diagnosis and preoperative evaluation. Although complete removal of the sinus tract usually resulted in good outcomes, regular postoperative follow-up remains important because recurrence may still occur.

The commonly accepted etiology of midline dermal sinuses is the ‘failure of separation theory, which suggests that during the critical period between 3 and 5 weeks of pregnancy, the separation between the neural ectoderm and the cutaneous ectoderm at the midline of the embryo is disrupted. This results in residual ectodermal tissue that persists between the skin and neural structures, leading to subsequent neurological abnormalities, such as tethered cord syndrome (5). In contrast to traditional midline dermal sinuses, the gluteal dermal sinus tract originates from a non-midline location. Its pathogenesis is due to the obstruction of “secondary neurulation” (11). This portion of the sinus tract does not extend into the lumbar region, and most terminate beside the fascia along the lower sacral spine (11). As a result, neurological symptoms are rare, and the condition typically presents as gluteal depression accompanied by recurrent infections. Additionally, while midline dermal sinuses are often associated with epidermoid cysts, paracentral dermal sinuses resemble epidermoid cysts (10, 15). A comparison of representative published studies on congenital gluteal or lateral dermal sinus tract is shown in Supplementary Table S1, highlighting differences in age at presentation, imaging strategy, lesion termination, surgical treatment, and prognosis.

Because the clinical presentation of congenital gluteal dermal sinus tracts is similar to that of fistula-in-ano and perianal abscesses (17–19), this condition is often misdiagnosed, and delayed diagnoses are common (mean time to diagnosis: 26.27 ± 28.44 months). Therefore, early detection and diagnosis are crucial. Imaging methods play an essential role in diagnosing this condition. Currently, MRI is the preferred method for evaluating congenital gluteal dermal sinus tracts because it provides detailed soft tissue contrast without ionizing radiation (20). Although CT is less effective than MRI in identifying the relationship between the fistula and the spinal canal, it can clearly show the position of the fistula within the dermis and fat layers and has a lower rate of missed diagnoses (11). Consistent with previous studies, our study shows that CT has high sensitivity for diagnosing gluteal dermal sinus tracts but lower specificity, making it prone to misdiagnosis. In contrast, MRI offers both high sensitivity and specificity, and is therefore recommended as the primary diagnostic tool. In addition to CT and MRI, ultrasound and fistulography are also commonly used in clinical diagnosis and treatment. Ultrasound is a convenient and effective imaging modality, often used for screening and providing clinical evidence to guide further CT and MRI examinations (14). In this study, all diagnosed patients underwent ultrasound screening in the outpatient setting, followed by CT and MRI for further evaluation. Fistulography helps determine the path and branches of the fistula, aiding in complete excision during surgery.

Complete surgical excision of the fistula is the established curative treatment for this condition (4, 10). In this study, all cases underwent complete excision of the fistula along its course during surgery. For posterior anal fistulas, excision should be as close to the fistula as possible to minimize damage to the sphincter. For lateral fistulas, the gluteus maximus muscle needs to be incised to fully expose the fistula. For cases with an associated cyst at the end of the fistula, the cyst should also be excised. Additionally, in cases with two openings (Type 3), it is important to verify whether they communicate at deeper levels. Methylene blue can be used as an optional tracer to detect their connection at deeper sites. In the cases in this study, the fistulas terminated in the anterior sacrum without reaching the spinal canal, but in some other cases, fistulas may connect to the spinal cord. In such instances, a neurosurgical consultation is recommended, and care must be taken to avoid injury to the spinal canal during surgery (8, 11). Large cavities left after fistula excision can be filled with a partial iliocostalis lumborum muscle flap (9).

This study has several limitations. First, it was a retrospective single-center study with a relatively small sample size, which may limit the generalizability of the findings; second, although long-term follow-up data were available, the follow-up duration was not completely uniform across all patients, and some cases had relatively limited follow-up. In future studies, we will continue follow-up and further stratify outcomes into short-term (<1 year) and long-term (≥1 year) follow-up groups to enable more reliable comparisons and draw more robust conclusions.

In summary, this study comprehensively described the clinical features, diagnostic evaluation, surgical management, and prognosis of 20 pediatric patients with congenital gluteal dermal sinus tract, and as a relatively large single-center series on this rare condition, it provides valuable clinical insights for the diagnosis and treatment of similar cases in other regions.

## Data Availability

The original contributions presented in the study are included in the article/Supplementary Material, further inquiries can be directed to the corresponding authors.

## Supplementary material

SUPPLEMENTARY TABLE S1 Comparison of published studies on congenital gluteal/lateral dermal sinus tract in children
